# The Effects of Embedded Skin Cancer Interventions on Sun-Safety Attitudes and Attention Paid to Tan Women on Instagram

**DOI:** 10.3389/fpsyg.2022.838297

**Published:** 2022-04-08

**Authors:** Jessica Gall Myrick, Katja Anne Waldron, Olivia Cohen, Carlina DiRusso, Ruosi Shao, Eugene Cho, Jessica Fitts Willoughby, Rob Turrisi

**Affiliations:** ^1^Donald P. Bellisario College of Communications, Pennsylvania State University, University Park, PA, United States; ^2^College of Health and Human Development, Pennsylvania State University, University Park, PA, United States; ^3^Ketchum, Inc., New York, NY, United States; ^4^Department of Communication Studies, The College of New Jersey, Ewing Township, NJ, United States; ^5^Edward R. Murrow College of Communication, Washington State University, Pullman, WA, United States

**Keywords:** social media, health campaigns, cancer communication, emotions, pride, attention

## Abstract

**Background and Objectives:**

Because of high skin cancer risks for young women, it is vital that effective interventions reach and influence this demographic. Visual social media platforms, like Instagram, are popular with young women and are an appropriate intervention site; yet, they also host competing images idealizing tan skin. The present study tested the ability of digital sun-safety interventions to affect self-control-related emotions and visual attention to subsequent tan-ideal images as well as sun-safety attitudes.

**Methods:**

Women were recruited from a large public Mid-Atlantic university in the United States. Participants (*N* = 120) were randomly assigned to view an appearance benefits intervention, a self-control emotions intervention, or a control message, each designed to look like an Instagram sponsored story. After self-reporting self-compassion and anticipated pride, participants then viewed seven pairs of Instagram posts featuring either tan or pale women while an eye tracker assessed visual attention. Finally, participants self-reported their responses to questions assessing sun-safety-related norms, efficacy, and attitudes.

**Results:**

A mixed design analysis of covariance revealed that women who first viewed the appearance benefits intervention story spent less time visually fixated on Instagram images of tan women than did those who viewed the self-control emotions intervention or control message (*p* = 0.005, $\eta_{p}^{2}$ = 0.087). Regressions also revealed interactions between the intervention conditions and feelings of anticipated pride on both visual attention and sun-safety attitudes.

**Conclusion:**

Sponsored stories on Instagram can promote sun-safety attitudes, depending on the emotional responses they generate. Additionally, sponsored interventions can affect subsequent visual attention.

## Introduction

Preventing skin cancer, which can be caused by exposure to ultraviolet (UV) light from the sun, is an urgent concern: each year in the United States, there are more new cases of skin cancer than cases of breast, prostate, lung, and colon cancer combined and is one of the most common cancers among young women under the age of 30 (American Cancer Society, 2017, 2021; Siegel et al., 2020). Because of higher skin cancer risks for young women compared to young men (Raimondi et al., 2020), it is vital that effective interventions reach and influence this demographic. Yet, the medium that is most popular with young women, social media (Pew Research Center, 2018), is also filled with content (user- and industry-generated) that promotes tan skin as the cultural ideal (Ricklefs et al., 2016; Banerjee et al., 2018; Waring et al., 2018). Although notable research has begun testing social media-based interventions to encourage young women to avoid UV exposure (Buller et al., 2022), research has not yet examined if those interventions prevent young women from also paying attention to the pro-tanning content that, contrary to the goal of interventions, promotes cancer-causing behaviors in young women.

As such, we need to develop theoretically sound sun-safety messages that can also capture the attention of college-age women, even amid a cacophony of pro-tanning messages on social media. The value of an intervention, no matter how theoretically sound, may quickly dissipate after women also view content promoting tan skin as the ideal. The purpose of this paper is, therefore, 3-fold: (1) test how well an established skin cancer intervention approach applies in the visual social medial context of Instagram; (2) compare this established intervention approach to an alternative one informed by the literature on positive psychology; and (3) integrate research on the role of visual attention to media content that contradicts the intervention’s purpose into this research area.

Notably, previous research has found that discussing the appearance harms of tanning, such as premature skin aging, and suggesting behavioral alternatives for improving one’s appearance (e.g., using make-up and spray tans) can effectively promote healthier skin-related behaviors (Sontag and Noar, 2017), in line with the Behavioral Alternatives Model (BAM) in the health behavior change literature, because appearance motivations are a strong predictor of tanning behavior (Hillhouse et al., 2008, 2017). Theoretically, these interventions have been shown to shape important psychological predictors of behavior change, like self-efficacy, norms, and attitudes (Hillhouse et al., 2000, 2016), with self-efficacy, norms, and attitudes important predictors of behavior based on the Theory of Planned Behavior (Ajzen, 2011).

Another approach to persuading young women to avoid skin-harming behavior is to apply lessons from positive psychology to social media interventions. Emotions motivate individuals to take action, with specific emotions associated with specific action tendencies, according to the Appraisal Theory of Emotions (Lazarus, 1991). Some positive emotions, like self-compassion and authentic pride, promote self-control in the face of temptation, helping people take the actions they need to achieve difficult goals (Williams and DeSteno, 2010; Valdesolo and DeSteno, 2014; DeSteno, 2018). Self-compassion involves viewing oneself with care and support when one is suffering (Neff, 2003b). Instead of getting upset for past failures (e.g., tanning that increased cancer risk or resulted in comically dark burns) or becoming defensive about those failures, the self-compassion literature suggests an approach whereby individuals are prompted to think of themselves with warmth, connection, and concern and to avoid harsh self-judgment (Raes et al., 2011). Self-compassion is associated with increased self-improvement motivation (Breines and Chen, 2012). Additionally, authentic pride (as opposed to hubris) can motivate individuals to persist in their goal pursuit, even in the face of obstacles (Williams and DeSteno, 2008, 2009). Anticipating pride about performing a healthy behavior has been shown to serve a self-regulatory function by promoting stronger behavioral intentions (Onwezen et al., 2014).

These emotions are theoretically promising for the context of sun-safety interventions because researchers have found that emotional expectations (e.g., tanning will feel good) are often better predictors of tanning behavior than are beliefs about the health threat, appearance benefits, or even social approval/disapproval (Noar et al., 2014; Carcioppolo et al., 2019). Therefore, effective social media interventions could promote positive emotional associations with avoiding tan skin to foster the pursuit of sun-safety behaviors.

Because appearance benefit interventions have been effectively used in print and website formats, we expect they will also help young women avoid focusing too much on subsequent tan-ideal content. Moreover, the previous work tying self-compassion and anticipated pride to self-control, suggestions the self-control emotion approach should do the same. However, it is unknown which approach will be most strongly related to the avoidance of visual temptation, leading to both a hypothesis and a research question:

*H1*: Interventions (appearance benefit and self-control) will predict decreased subsequent visual attention to tan women on Instagram compared to the control condition.

RQ1: Which intervention type (appearance benefit and self-control) will be associated with less subsequent visual attention to tan women on Instagram?

While previous interventions have assessed outcomes grounded in the Theory of Planned Behavior, emotional responses, particularly positive emotions, are understudied in this area but should, theoretically, motivate individuals to pursue health-related goals, leading to another hypothesis:

*H2*: Emotional responses to the intervention (i.e., self-compassion and anticipated pride for future sun-safety behavior) will be negatively related to subsequent visual attention to tan women on Instagram.

However, it is less clear how the type of intervention and emotional responses may interact with each other to shape subsequent attention to tan images on social media, leading to a second research question:

RQ2: Will the type of intervention and self-control-oriented emotional responses to it (self-compassion and anticipated pride) interact to affect subsequent visual attention to tan women on Instagram?

It is likely, though, that the effects of the interventions and subsequent emotions operate alongside previously established psychosocial predictors of attitudes, leading to two final hypotheses:

*H3*: Appearance motivations, social norms, and self-efficacy will be positively related to sun-safety attitudes.

*H4*: Emotional responses to the intervention (i.e., self-compassion and anticipated pride for future sun-safety behavior) will be positively related to sun-safety attitudes.

Finally, research in this area of intervention effects has relied heavily on self-reports and is yet to integrate biometric markers of attention, leading to a final research question:

RQ3: Will visual attention to subsequent images of tan women predict sun-safety attitudes?

In sum, the present study offers an initial feasibility test comparing two theoretically sound intervention types in the context of a highly visual social media platform while also integrating biometric assessments of visual attention to competing content in the same platform as an additional outcome to consider when weighing the effectiveness of such interventions at shifting attitudes about skin health behaviors.

## Materials and Methods

### Participants

Students (*N* = 120) ages 18–22 years enrolled at a large public university in the mid-Atlantic United States were recruited to participate in a study (a three-condition experiment) about women and health messages. Potential participants were drawn from a list of all female students on the main university campus.

### Procedures

**Figure 1:**
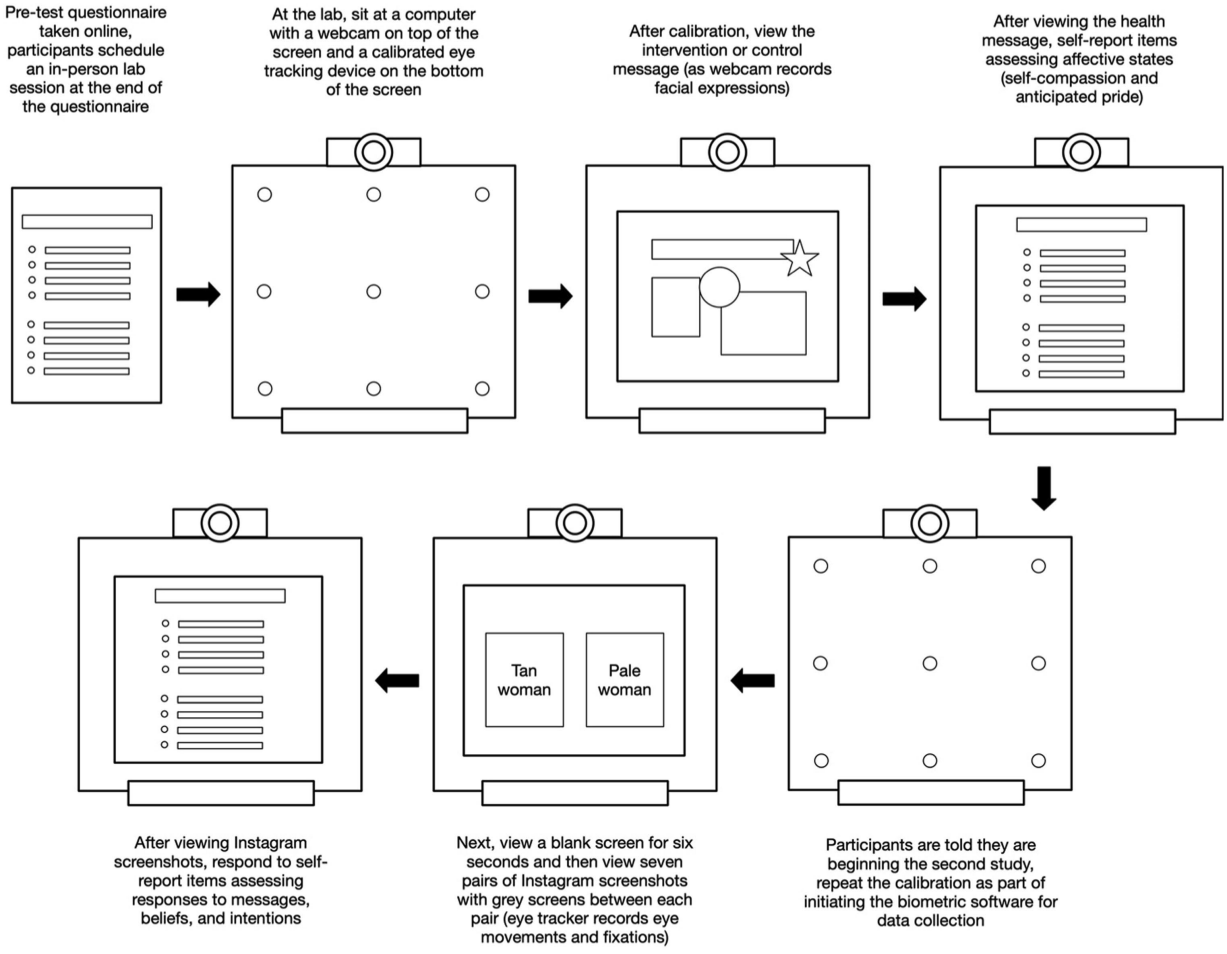
Procedures overview.

Sixty women participated in the study in April of 2019, and another 60 took part in September of 2019 (see Figure 1; *N* = 120). Prior to the main study, participants completed an online questionnaire to provide demographics, skin type information, and incentive preferences. At their later laboratory appointment, after providing consent, participants were seated at a chair in front of a computer with a webcam and a Tobii X2-60 eye-tracking device (integrated *via* iMotions software). They were told they were participating in two separate studies, one examining responses to health messages and the second analyzing responses to common media messages. Participants initially did a calibration activity to ensure the eye tracker was functioning and then began the study. Participants viewed either an intervention or control message designed to look like an Instagram story. Next, they took an online questionnaire to report their responses to the intervention message.

Then, they were told they would begin the second study about media messages. Participants redid the calibration exercise and then viewed a blank grey screen for 6 s before viewing seven sets of paired Instagram images featuring one tan and one pale woman. Next, participants responded to a Qualtrics questionnaire to report their beliefs and attitudes. Participants received $40 USD in the form of either monetary credit on their university identification card or an Amazon.com gift card. A university Institutional Review Board approved all procedures.

### Intervention Materials

The intervention materials are designed to look like Instagram stories, which are a platform feature that allow users to post a series of photos or videos, often edited with text overlays, on their account for their followers to view. Each photo or video slide can last for up to 15 s. The slides automatically advance to the next one as soon as the previous one’s time has elapsed (like to an automated slide show). The intervention messages and control message each included 10 slides featuring relevant text and images, each appearing on screen for 15 s (2 min 30 s total). See Table 1 for sample text from the treatment groups (the control condition features slides listing health-related resources available to students on campus, like medical clinics, counseling, and fitness facilities). Focus groups were conducted to help refine our initial materials. The slides used colors and fonts available *via* Instagram to increase the ecological validity of the materials.

**Table 1:** Sample intervention text.

Appearance focused intervention	Self-control emotions intervention
Have you ever tanned or laid out in the sun without sunscreen?	Have you ever tanned or laid out in the sun without sunscreen?
Researchers have learned that exposure to ultraviolet radiation (sunlight) causes skin cancer and 90% of skin aging.	Researchers have learned that exposure to ultraviolet radiation (sunlight) causes skin cancer and 90% of skin aging.
Things to do to prevent skin aging: 1. Avoid laying out in the sun to tan, 2. Use sunscreen every day, 3. Cover your skin, 4. Try alternative beauty approaches. We’ll provide tips on how to do these…	That’s okay. You’re human. Most everyone has gotten too much sun before. We are all human. You can’t change the past, and you can’t always avoid the sun, but you can learn and grow.
1. Laying out in the sun causes skin aging. Skin aging = wrinkles, sun spots, and leathery-looking skin. Your skin doesn’t forget. A healthy approach is laying out in the sun less than once a month.	Things to do to be kind to your skin: 1. Avoid laying out in the sun to tan, 2. Use sunscreen every day, 3. Cover your skin, 4. Embrace your future health. We’ll provide tips on how to do these…
4. Try alternative beauty approaches. Check out sunless tanning options. Get the look without the wrinkle-causing UV rays with spray tans. Or, try at-home sunless tanning products (sprays, creams, bronzers).	1. Avoid laying out and keep your skin safe. Be kind to yourself by being kind to your skin. You deserve to be healthy, now and in the future. A healthy approach is laying out in the sun less than once a month.
In conclusion… If you want to look good and be healthy, avoid tanning. Instead, use SPF 15+ sunscreen, reapply after 2 hours, cover up, try beauty alternatives	4. Embrace your future health. Confidence looks good on you. Check out other activities that make you feel strong and confident. Try physical activity or spending time in nature. Hang out with friends who support your health and goals.
	In conclusion… Be kind to yourself and your skin. You deserve it. Remember, use SPF 15+ sunscreen, reapply after 2 hours, cover up, be proud of your efforts to take care of yourself!

### Instagram Images

Fifty screenshots of actual Instagram images were compiled by searching through publicly viewable Instagram posts with tags related to tanning (e.g., #tan, #tanning, #sun, and #sunbathing), 25 featuring tan women and 25 featuring women with pale skin. A pretest with 39 female participants, recruited from Amazon’s Mechanical Turk and who viewed and rated all 50 Instagram posts, allowed us to form seven pairs of Instagram posts for use in the main study that were perceived as equally attractive and likeable where one woman had tan skin and the other had pale skin.

Participants viewed each pair of images for 10 s. They were shown side-by-side, following the procedures used by Marquart et al. (2016). After seeing a pair, participants then saw a slide with a grey background featuring a crosshair (like a plus sign) in the middle to help reorient visual attention to the center of the screen (this avoids locational bias in where visual attention begins on subsequent slides). Three random orders of the seven pairs were created and presented randomly to participants to guard against order effects. These random orders included different versions of the pairs so that sometimes the tan woman was on the left and sometimes she was on the right. The presenting of multiple images on a screen at the same time was employed to help approximate real-world social media use. While using Instagram, people do not typically view any one image in isolation for very long. Instead, they see multiple images in a row, and they make (largely implicit) decisions about where to direct their visual attention.

### Measures

Skin type was assessed during the pretest with a single item (Fitzpatrick, 1988) where individuals chose which of the following six options (coded 1 through 6) best described their skin type: always burn, never tan; usually burn, tan with difficulty; sometimes mild burn, tan about average; rarely burn, tan with ease more than others; rarely or never burn, my skin is brown; or rarely or never burn, my skin is black (*M* = 3.25, SD = 0.98).

Appearance motivations to tan were assessed during the pretest with five items (Cafri et al., 2008) measured on a scale from 1 (strongly agree) to 7 (strongly disagree): “How I look is important to me”; “It is important that others view my physical attractiveness positively”; “I would do whatever it takes to look good”; “It is important that I always look good”; and “I spend what others consider a large amount of time on my appearance daily” (*M* = 3.90, SD = 1.21, *α* = 0.89).

Self-compassion was assessed after exposure to either an intervention message or a control message and measured with three items adapted from Neff (2003a). Participants were asked “After viewing the message, please indicate the extent to which you currently feel” and then given three statements (patient toward myself, kind to myself, and tender toward myself) on a 1 (strongly disagree) to 7 (strongly agree) scale (*M* = 5.11, SD = 1.23, *α* = 90).

Anticipated pride was assessed after exposure to either an intervention message or a control message and assessed by asking participants to respond to the following prompt, adapted from previous work (van der Schalk et al., 2012): “Imagine that you are about to go outside on a warm sunny day, and you decide to protect your skin beforehand (you apply sunscreen before, or you wear protective clothing). How would you feel after protecting your skin?” They then rated their responses on a 1 (strongly disagree) to 7 (strongly agree) scale for the items proud, accomplished, confident, satisfied, and worth-while (*M* = 4.61, SD = 1.48, *α* = 0.90).

The total fixation time on all tan images was captured *via* the eye tracker while viewing the seven pairs of Instagram screenshots (*M* = 10,345.69 milliseconds; SD = 8,162.26 s).

Norms were assessed after viewing the Instagram images with six responses, adapted from Rimal and Real (2005), on a scale from 1 (strongly disagree) to 7 (strongly agree), to the following statements: “Most women my age tan outdoors”; “The most popular women my age tan outdoors”; “It is appropriate to tan outdoors”; “Society in general considers outdoor tanning an acceptable behavior”; “Most women my age in general consider outdoor tanning acceptable”; and “Most people in general consider outdoor tanning an appropriate behavior” (*M* = 5.71, SD = 0.82, *α* = 0.72).

Efficacy was assessed after viewing the Instagram images and measured with six items adapted from Noar et al. (2015) on a scale from 1 (not at all confident) to 7 (extremely confident) in response to asking participants to indicate how confident they are that they could perform the following behaviors when it is sunny: “Use sunscreen whenever you are out in the sun for more than 15 min”; “Use sunscreen when no one else you are with is using sunscreen”; “Use sunscreen even if you do not like how it feels”; “Stay in the shade when all your friends are enjoying themselves in the sun”; “Cover up with protective clothing even when it is hot outside”; and “Avoid going outside in the sun during midday hours” (*M* = 3.60, SD = 1.33, *α* = 0.81).

Sun-safety attitudes were adapted from Hillhouse et al. (2017) and assessed after viewing the Instagram images. Participants responded on a scale from 1 (strongly disagree) to 7 (strongly agree) to the following five statements that were based on behaviors mentioned in the interventions: “I feel good about laying out in the sun less than once a month”; “I feel good about wearing a sunscreen of SPF15 or higher every day”; “I feel good about re-applying sunscreen about every 2 h”; “I feel good about wearing a cover-up or other clothing when outside in the sun”; and “I feel good about finding alternative behaviors besides outdoor tanning” (*M* = 4.05, SD = 1.22, *α* = 0.68). See Supplementary Material for correlations between variables in the analyses.

## Results

H1 predicted the interventions would decrease time spent fixated on subsequent social media images of tan women, and RQ1 asked which intervention would have the strongest effect. To determine which variables should serve as covariates in the analysis, we first ran bivariate correlations between the total summated time spent fixated on all seven of the tan images with participant skin type, data collection wave (April or September), and two dummy coded variables representing the three possible orders of presentation of the Instagram posts. Skin type was the only significant variable (*r* = −0.34, *p* < 0.001; meaning participants with skin that easily burns were less likely to spend time fixated on the tan images) and was, therefore, retained as a covariate.

Because participants viewed multiple images, a mixed design analysis of covariance (ANCOVA) was used with the seven tan images as the within-subjects factor, intervention condition (appearance benefits, pride and self-compassion, or control) as the between-subjects variable, skin type as the covariate, and fixation time on the images of tan women as the dependent variable.

Mauchly’s test of sphericity was significant (*p* < 0.001, Greenhouse-Geisser estimate = 0.730). As such, the Greenhouse-Geisser correction was used on degrees of freedom in interpreting effects. The test of within-subjects effects was not significant for the tan images, *F*(4.380, 503.68) = 0.859, *p* = 0.497, $\eta_{p}^{2}$ = 0.007, indicating that participants did not spend significantly different amounts of time fixated on any one of the seven images of tan women. Additionally, there was not a significant interaction between the particular tan image seen and intervention condition, *F*(8.760, 503.68) = 1.625, *p* = 0.107, $\eta_{p}^{2}$ = 0.027, meaning that effect of the intervention condition did not vary for different tan images. Additionally, there was not a significant interaction between the tan images and skin type, *F*(4.380, 503.68) = 0.472, *p* = 0.773, $\eta_{p}^{2}$ = 0.004, suggesting that people with different skin types did not respond differently to each tan images.

**Figure 2:**
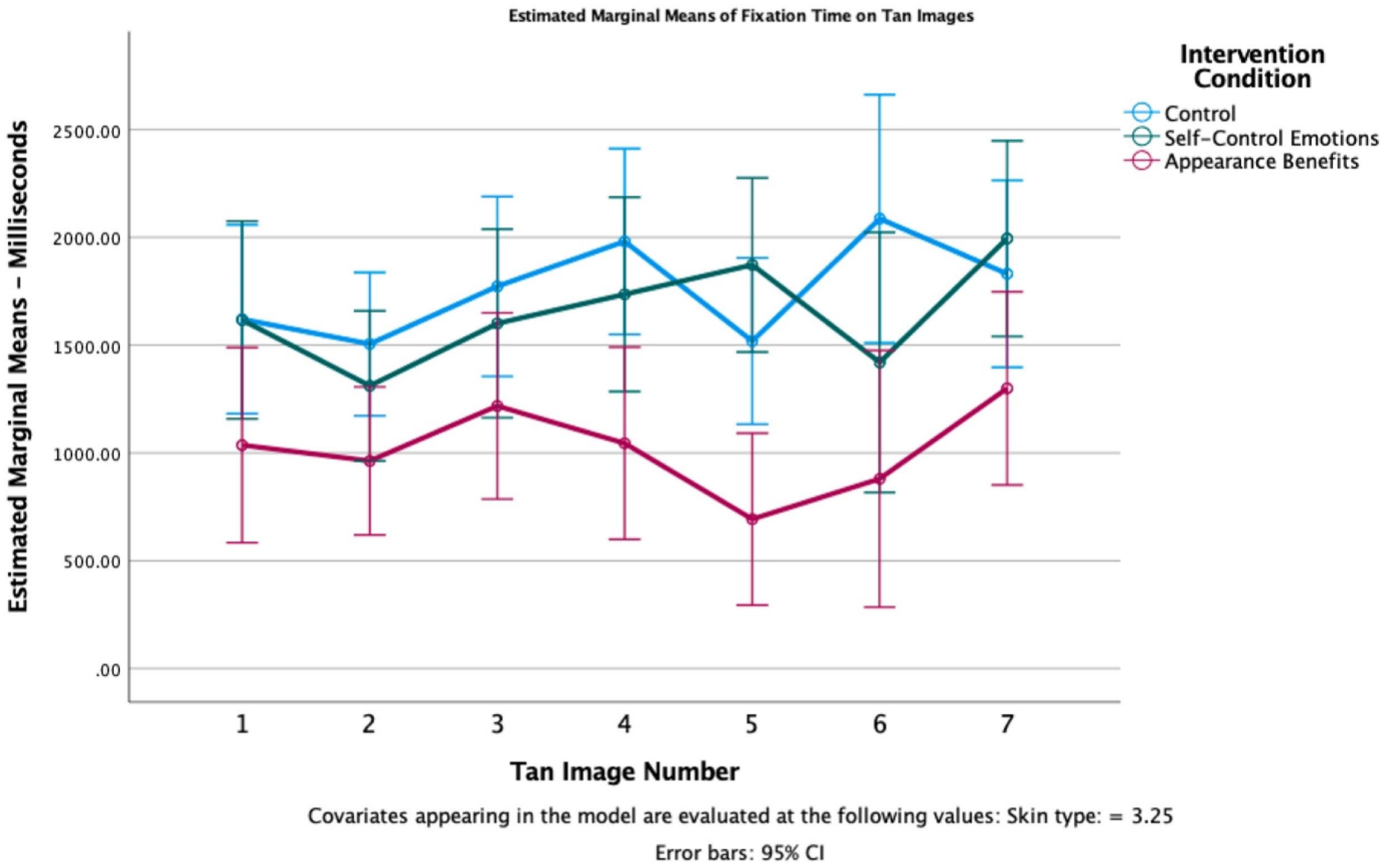
Fixation time on the tan images by condition for each pair of Instagram screenshots.

The test of the between-subjects effect of the intervention condition on time spent fixated on tan images was significant: *F*(2, 115) = 5.484, *p* = 0.005, $\eta_{p}^{2}$ = 0.087 (see Figure 2). For all seven pairs of Instagram images, participants who first viewed the appearance benefits intervention spent less time visually fixated on the image of the tan woman than did people who viewed the self-control emotions intervention or the control message, partially supporting H1.

H2 (self-control emotions would be negatively related to visual attention to tan women) and RQ2 (the interaction of emotions and intervention type) asked about the role of self-compassion and anticipated pride responses to the intervention in potentially shaping the effects of the intervention on subsequent attention to images of tan women. A stepwise regression was run to address them. We retained skin type as a control variable prior to introducing the main effects of condition (a dummy coded variable for the appearance benefit condition and a dummy coded variable for the self-control emotions condition), self-compassion, and anticipated pride. In the second block, four interaction terms were entered between the two condition variables and the two emotion variables (see Table 2). Model 1, with only the main effects, was significant: *F*(5, 113) = 6.05, *p* < 0.001, *R^2^* = 0.21. This model revealed significant main effects of skin type (*β* = −0.40, *p* < 0.001) and the appearance benefits condition (*β* = −0.30, *p* = 0.003), but none for the main effect of emotions, meaning H2, was not supported. Model 2, the block with the interactions, also approached significance: Δ*F*(4, 109) = 2.43, *p* = 0.052, Δ*R^2^ =* 0.07. In Model 2, the interaction between the appearance benefits condition and anticipated pride (*β* = 1.00, *p* < 0.001) was significant. That is, for participants who viewed the appearance benefits intervention, as anticipated pride increases, there is more visual attention paid to tan women. However, when participants do not view the appearance benefits intervention, as anticipated pride increases, less visual attention is paid to tan women.

**Table 2:** Standardized beta coefficients of the stepwise regressions.

Time fixated on tan images			Sun-safety attitudes		
	Model 1	Model 2		Model 1	Model 2
Predictors	*β*	*β*	Predictors	*β*	*β*
Skin type	−0.40^***^		Wave	0.12	
Self-compassion	0.16		Efficacy	0.50^***^	
Anticipated pride	−0.07		Self-compassion	−0.10	
SCE condition	−0.02		Anticipated pride	0.28^***^	
AB condition	−0.30^**^		SCE condition	0.07	
SCE X self-comp		−0.12	AB condition	0.04	
SCE X ant-pride		0.50	Time Fixated	−0.04	
AB X self-comp		−0.15	SCE X self-comp		0.62
AB X ant-pride		1.00^**^	SCE X ant-pride		−0.81^*^
			AB X self-comp		0.49
			AB X ant-pride		−0.41

*SCE*, *Self-control emotions; AB, Appearance benefits*.

* *p < 0.05;*

** *p < 0.01;*

*** *p < 0.001.*

H3 predicted that appearance motivations, social norms, and self-efficacy would predict attitudes while H4 predicted emotional responses would do the same, while RQ3 asked if visual attention would also predict attitudes. To address these issues, we ran a stepwise linear regression predicting with sun-safety attitudes as the dependent variable. We started by running bivariate correlations with the same potential control variables tested prior to the analyses predicting fixation time (participant skin type, data collection wave, and dummy codes for the presentation order of the Instagram posts) with sun-safety attitudes. Only wave and efficacy were significantly correlated and kept in the final analysis in the first block. We also included the main effects of self-compassion, anticipated pride, appearance benefits intervention, self-control emotions intervention, and time spent fixated on the tan woman images in the first block. In the second block, we included the four interaction terms (see Table 2).

Model 1, with the main effects, was significant: *F*(7, 111) = 14.52, *p* < 0.001, *R^2^* = 0.48. In this model, efficacy (*β* = 0.50, *p* < 0.001) and anticipated pride (*β* = 0.28, *p* < 0.001) were significant predictors of positive attitudes; as such, H3 and H4 were only partially supported. Additionally, Model 2, the interactions block, was not significant: Δ*F*(4, 107) = 1.86, *p* = 0.122, Δ*R^2^ =* 0.03. In Model 2, the interaction between the self-control emotions condition and anticipated pride was significant (*β* = −0.81, *p* = 0.011). When people see the self-control intervention, as anticipated pride increases, there is more positive sun-safety attitudes. But, when they do not see the self-control emotions intervention, as anticipated pride increases, they report lower attitudes.

## Discussion

By combining eye tracking and self-report assessments, we were able to determine that different interventions and emotional responses to them can shape attention and attitudes. In a world where young women spend a lot of time on social media, where there are many images of the tan-ideal, it is crucial to consider how to best promote attentional, attitudinal, and, eventually, behavioral change in the midst of content that may undermine intervention efforts.

In the highly visual context of Instagram, our data suggest that using an appearance benefits-focused intervention lessens women’s gaze on the tan ideal. However, this finding comes with a caveat: If participants also report feeling greater anticipated pride for performing sun-safety behaviors in the future, then the appearance benefits story actually promotes greater visual attention to tan women. It could be that when the appearance benefits condition generates pride for future healthier behaviors, young women then scrutinize others who are clearly not performing sun-safety behaviors. Additional interviews and focus groups could help clarify the nuances of the quantitative findings.

Anticipated pride also played a role in understanding attitudinal effects. If participants viewed the self-control intervention and felt higher levels of anticipated pride, then they reported more positive sun-safety attitudes. However, without seeing the self-control emotions intervention, stronger feelings of anticipated pride resulted in less positive attitudes. This suggests interventions focusing on self-control emotions would be wise to promote pride, while other interventions with other goals may want to avoid sparking that emotion. Together, these findings suggest that anticipated emotional responses related to a health behavior are important variables that could be included in future models of planned behavior change, particularly since many interventions ask participants to explicitly think about their future (e.g., have healthier and prettier skin).

These findings are not without their limitations. The small sample was isolated to one university and women and improvement in internal reliability is needed for some measures. We also did not track longitudinal behavior change after our interventions. Give the novelty of the approach combining biometrics and self-report responses to interventions, there are benefits to continuing this line of work. Future research could combine longer data collection periods with biometric assessments of emotional responses, too, to better capture dynamic changes in responses to social media, which is also a dynamic environment.

These results point to the need to understand self-control-related emotional responses and when they might facilitate attitudinal change after viewing social media and when they may, instead, stymie efforts to motivate young women to avoid UV exposure. Additional research is needed to probe the complex interplay of health messaging, visual attention, and attitudes resulting from social media use, but the present data provide an important starting point for this effort.

## Data Availability Statement

The raw data supporting the conclusions of this article will be made available by the authors, without undue reservation.

## Ethics Statement

The studies involving human participants were reviewed and approved by the Pennsylvania State University Institutional Review Board. Written informed consent for participation was not required for this study in accordance with the national legislation and the institutional requirements.

## Author Contributions

JM participated in conceptualization, design, data collection, data analysis, and writing. KW participated in conceptualization, data collection, data cleaning, and writing. OC and RS participated in data collection and writing. EC participated in data collection, data cleaning, and writing. JW participated in conceptualization, design, and writing. RT participated in conceptualization, design, data analysis, and writing. All authors contributed to the article and approved the submitted version.

## Funding

This research was funded by the Social Science Research Institute at Pennsylvania State University.

## Conflict of Interest

CD joined Ketchum, Inc. after data collection was complete.

The remaining authors declare that the research was conducted in the absence of any commercial or financial relationships that could be construed as a potential conflict of interest.

## Publisher’s Note

All claims expressed in this article are solely those of the authors and do not necessarily represent those of their affiliated organizations, or those of the publisher, the editors and the reviewers. Any product that may be evaluated in this article, or claim that may be made by its manufacturer, is not guaranteed or endorsed by the publisher.
