# Supplementary material for: The Effects of Embedded Skin Cancer Interventions on Sun-Safety Attitudes and Attention Paid to Tan Women on Instagram
Source: Front Psychol. 2022 Apr 8;13:838297. doi: 10.3389/fpsyg.2022.838297 (PMC9029166; doi:10.3389/fpsyg.2022.838297)
Supplement: Supplementary file 1 [file Table_1.docx]

Screenshots from the Interventions Conditions and Text From the Control Condition

Sample content from the Self-Control Emotions Intervention


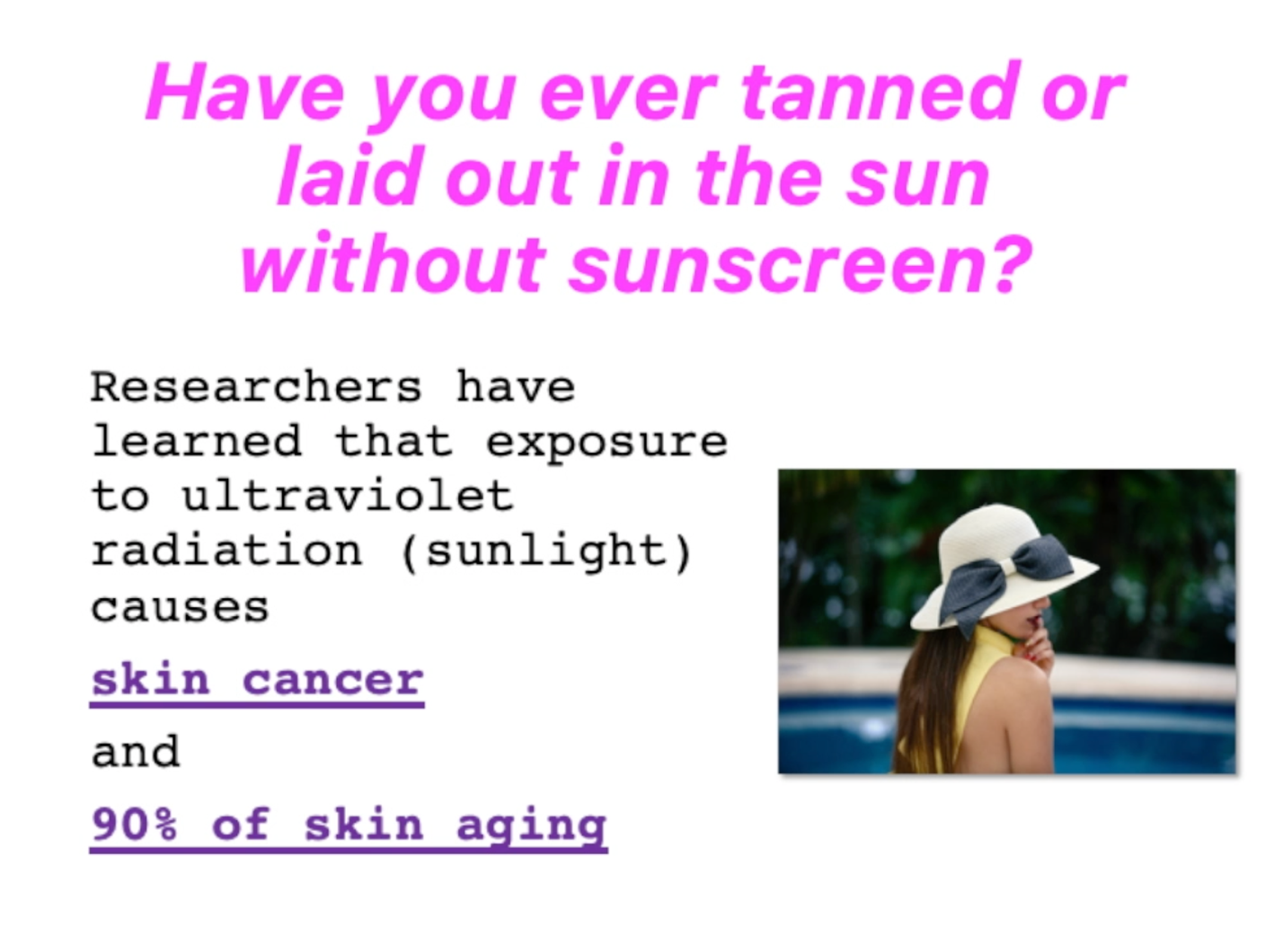

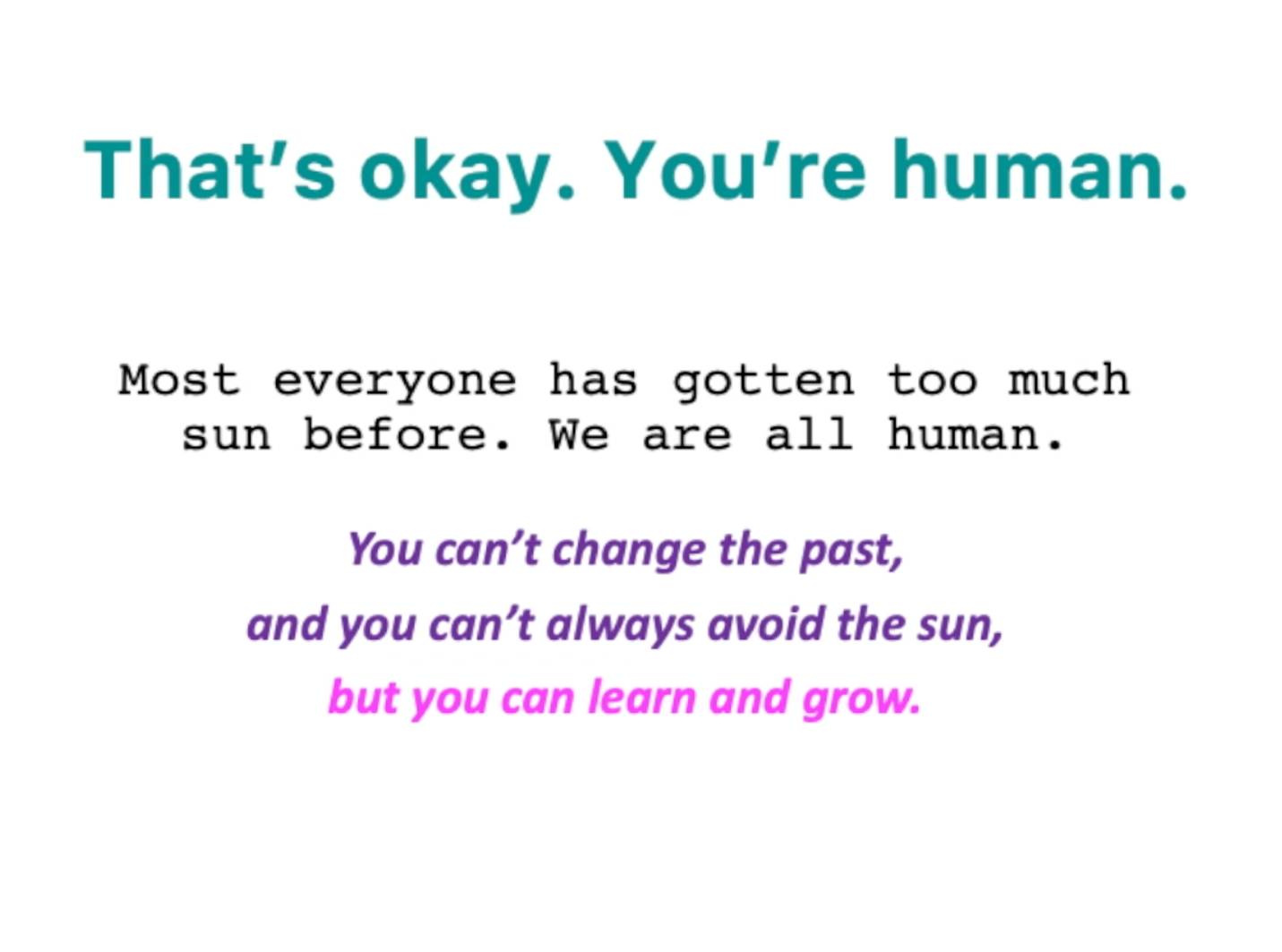

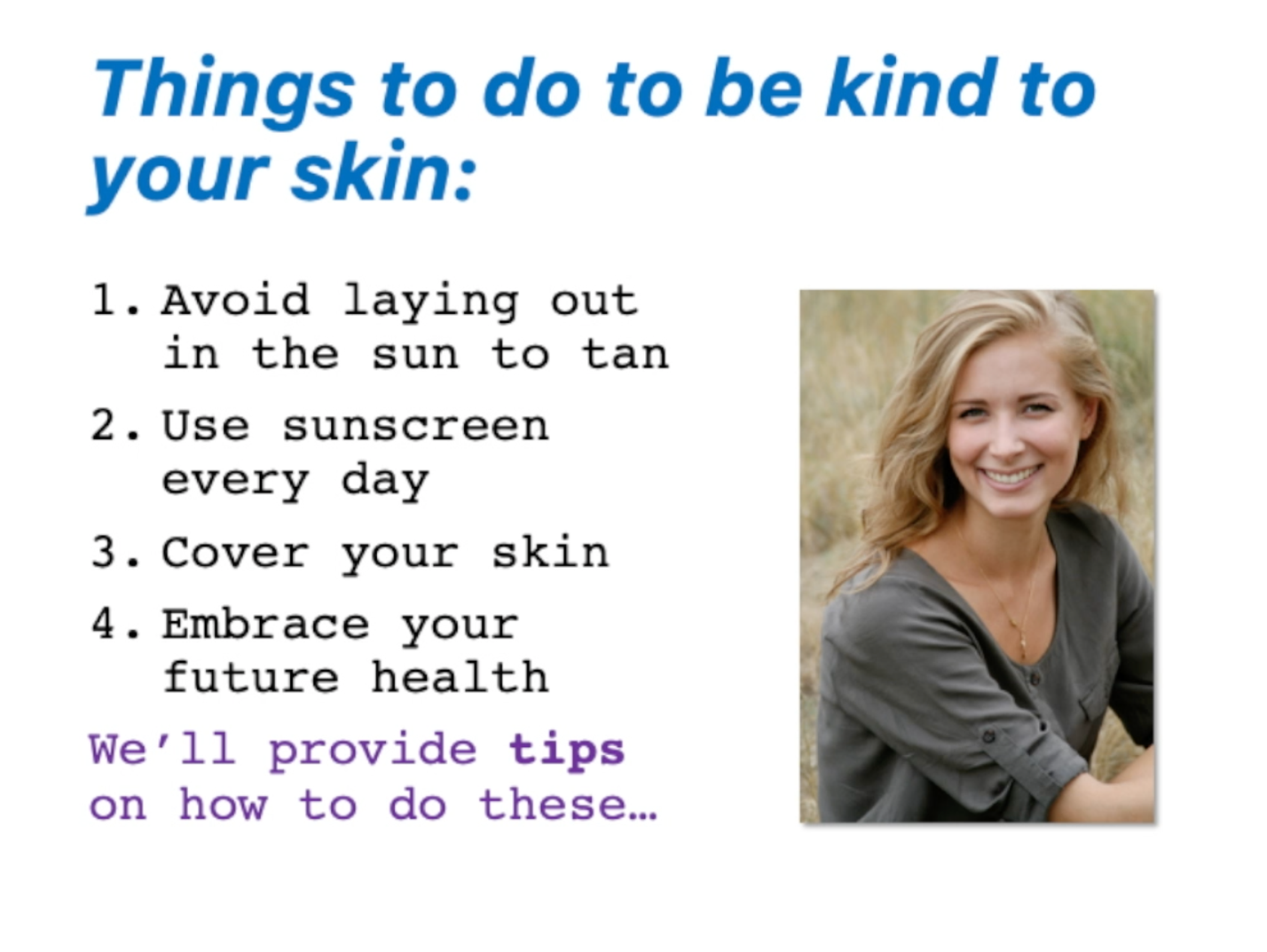

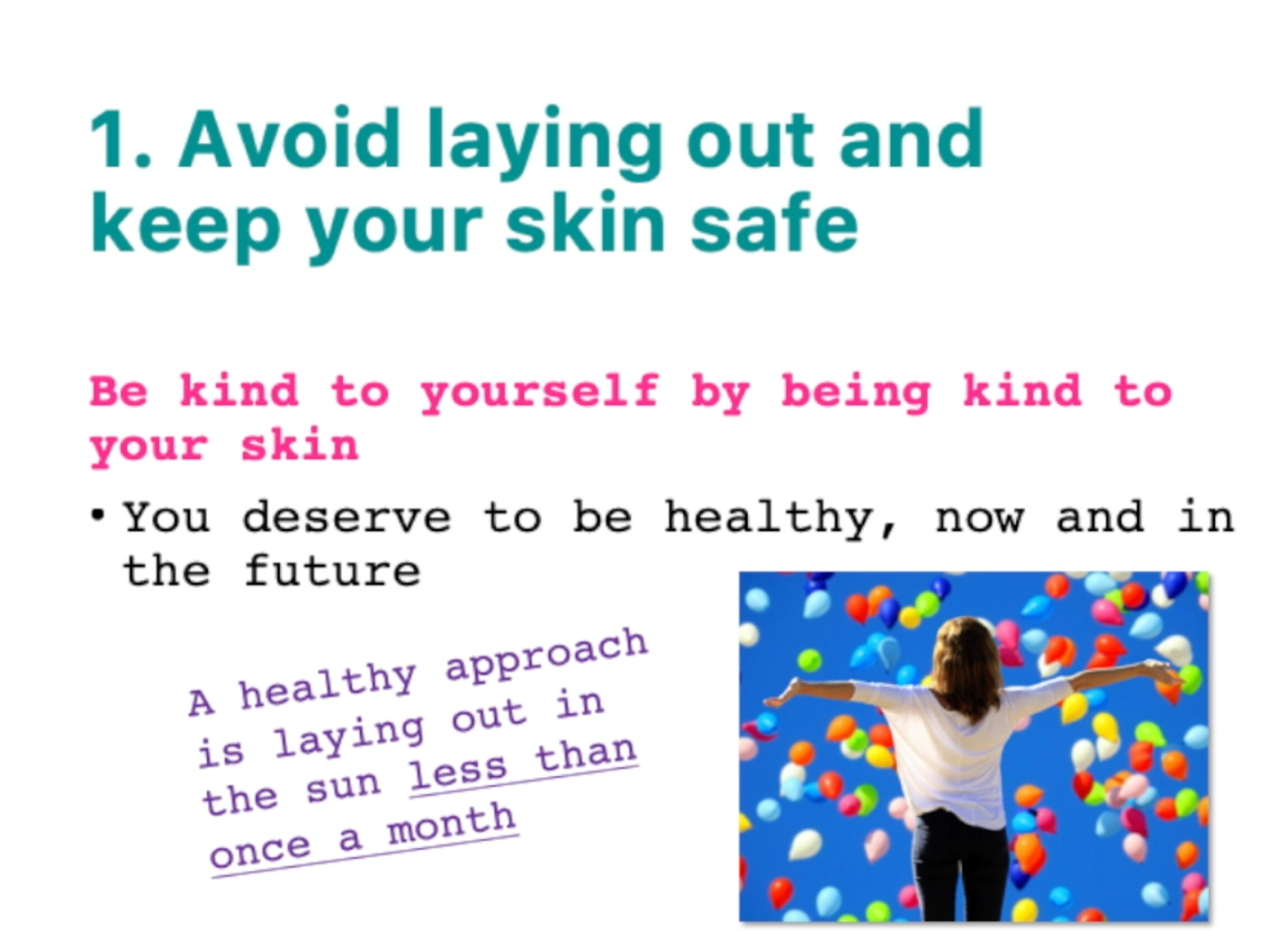

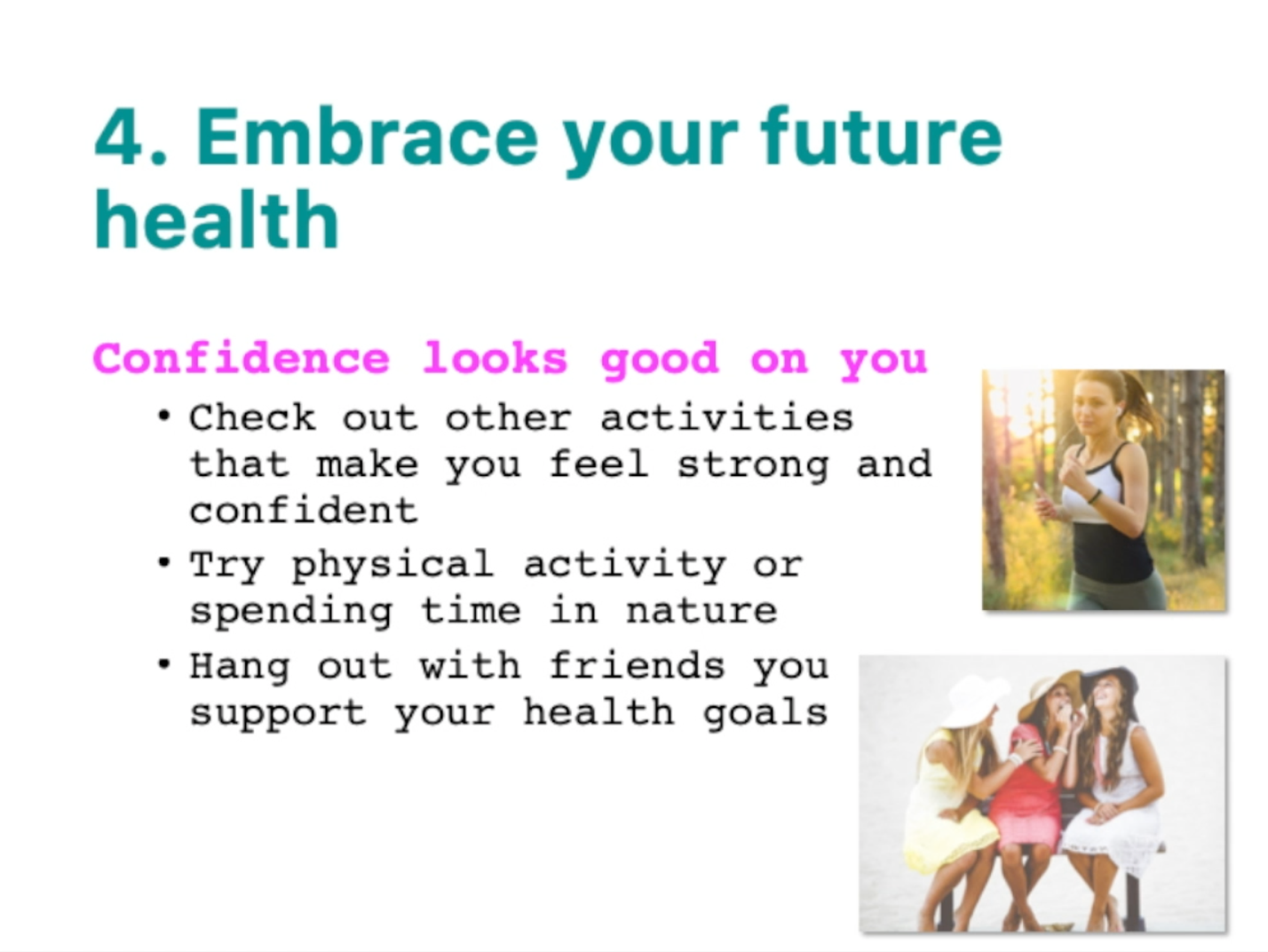

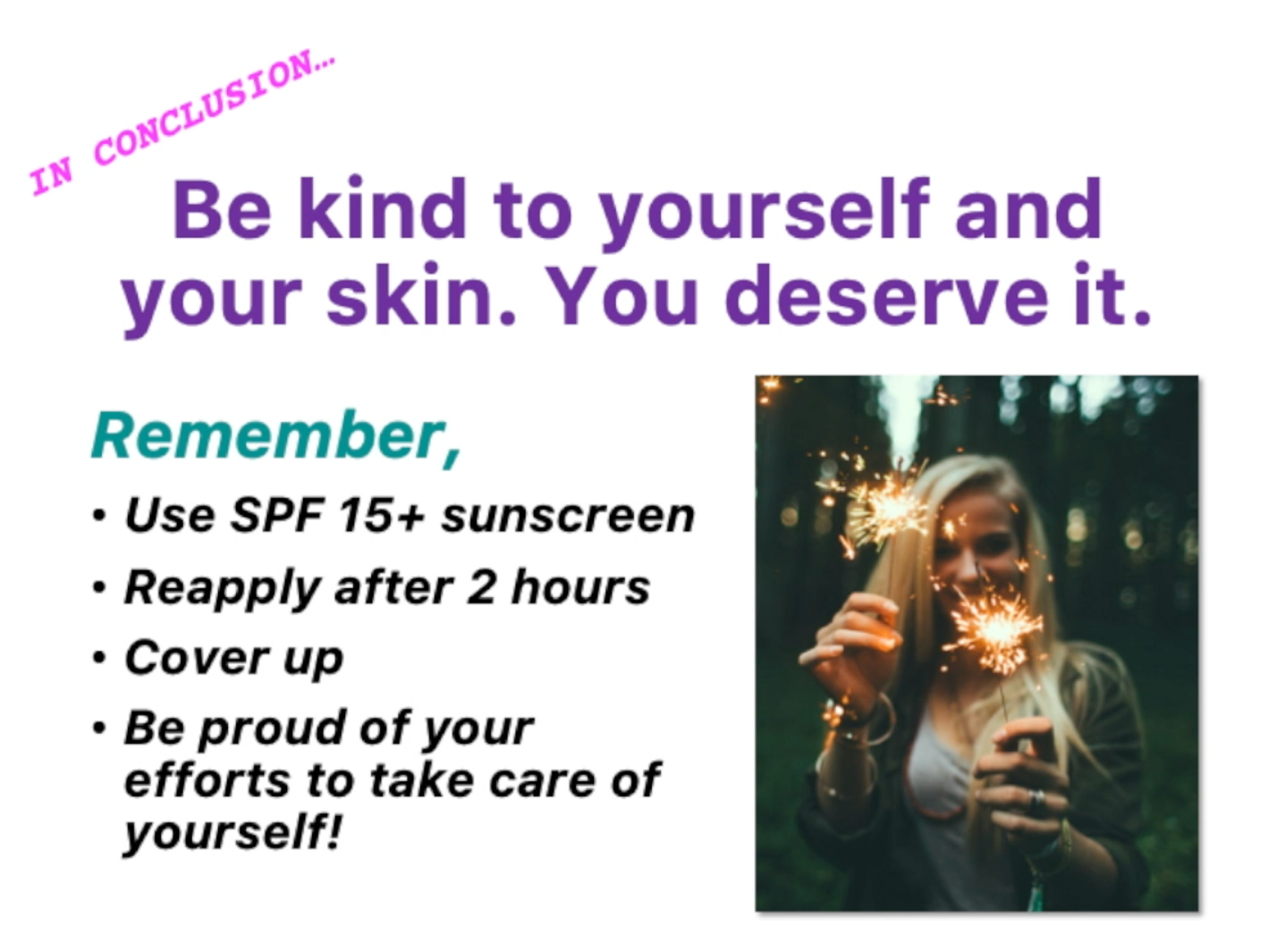


Sample content from the Appearance Benefits Intervention


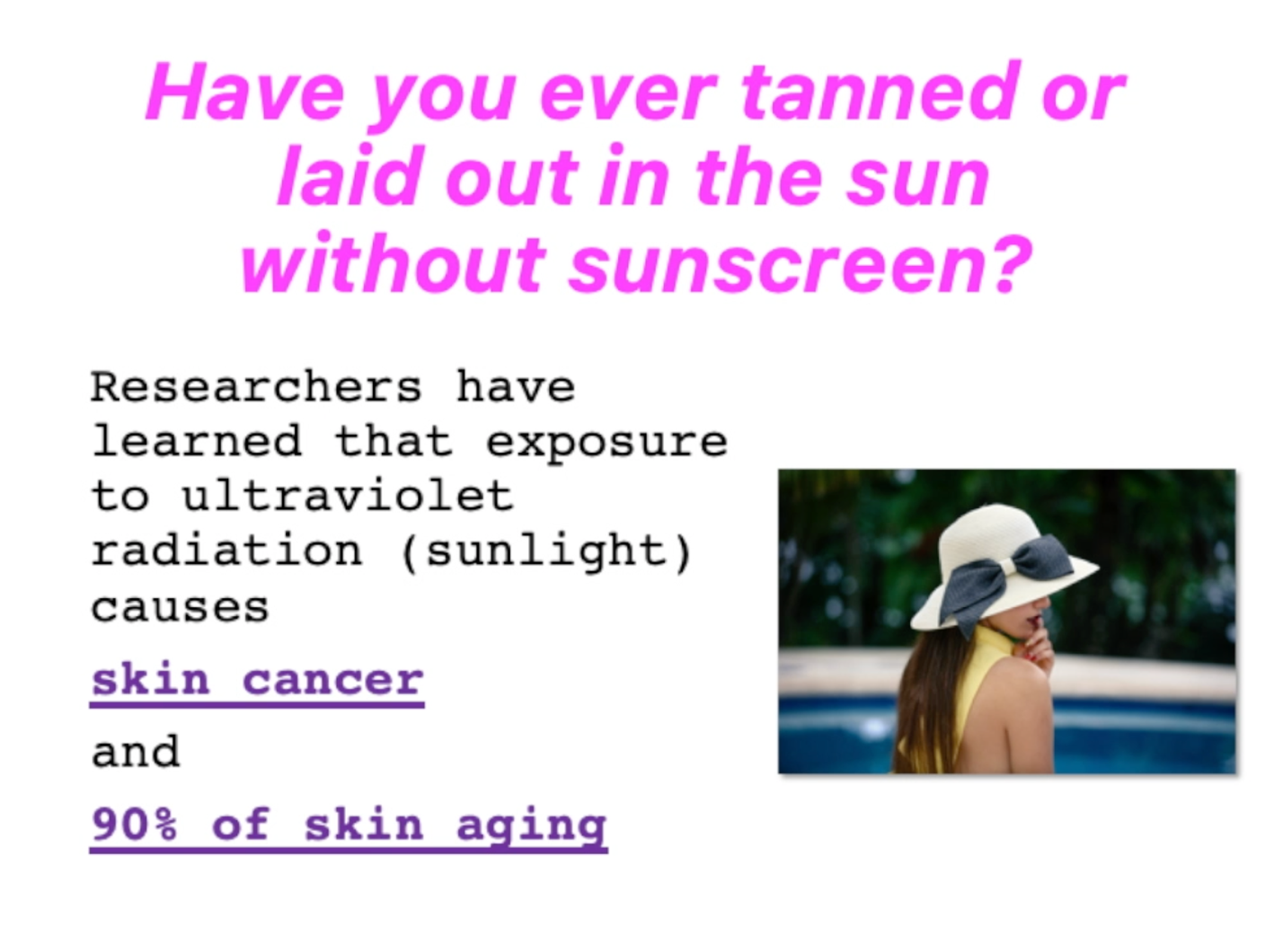


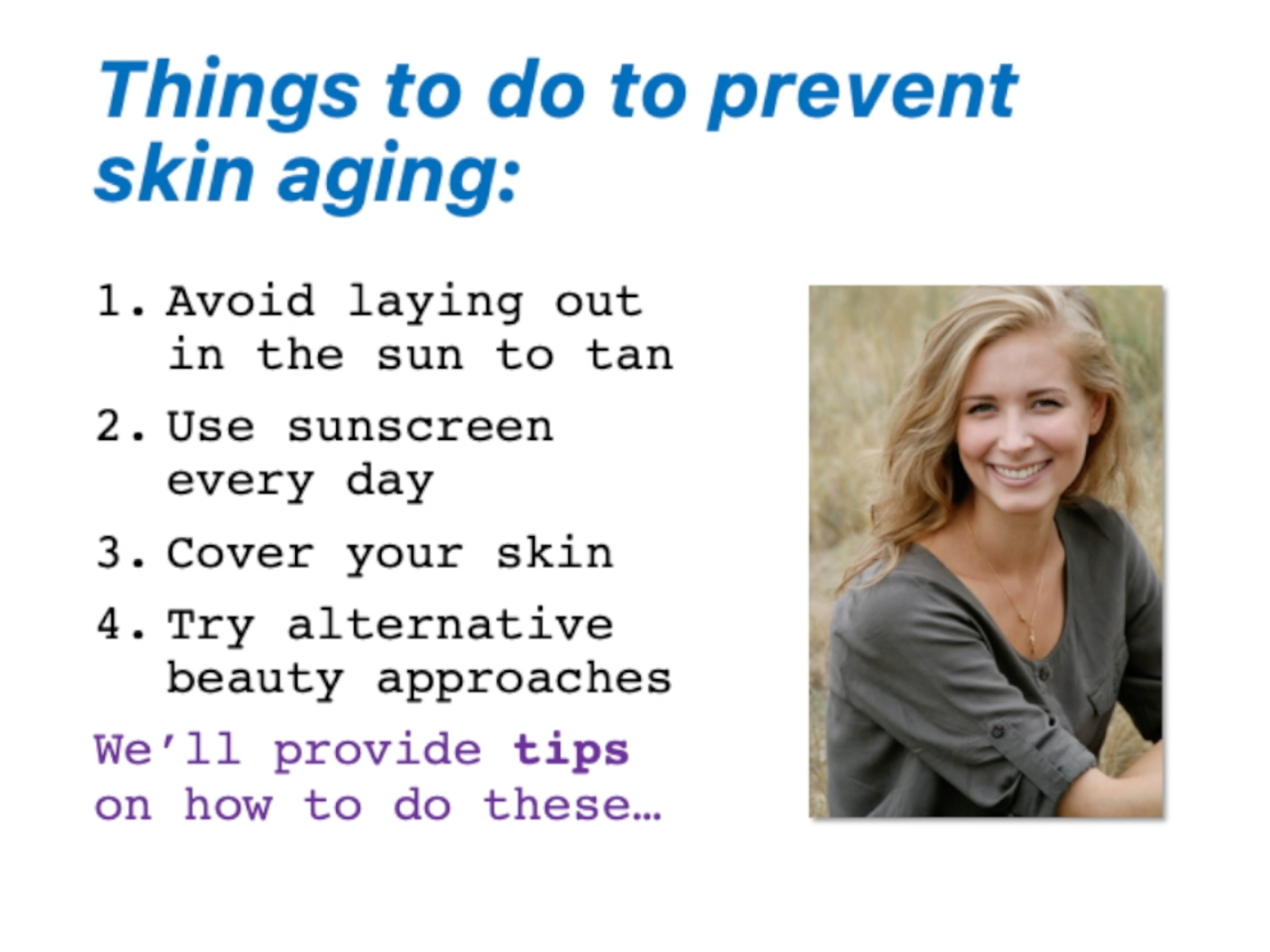

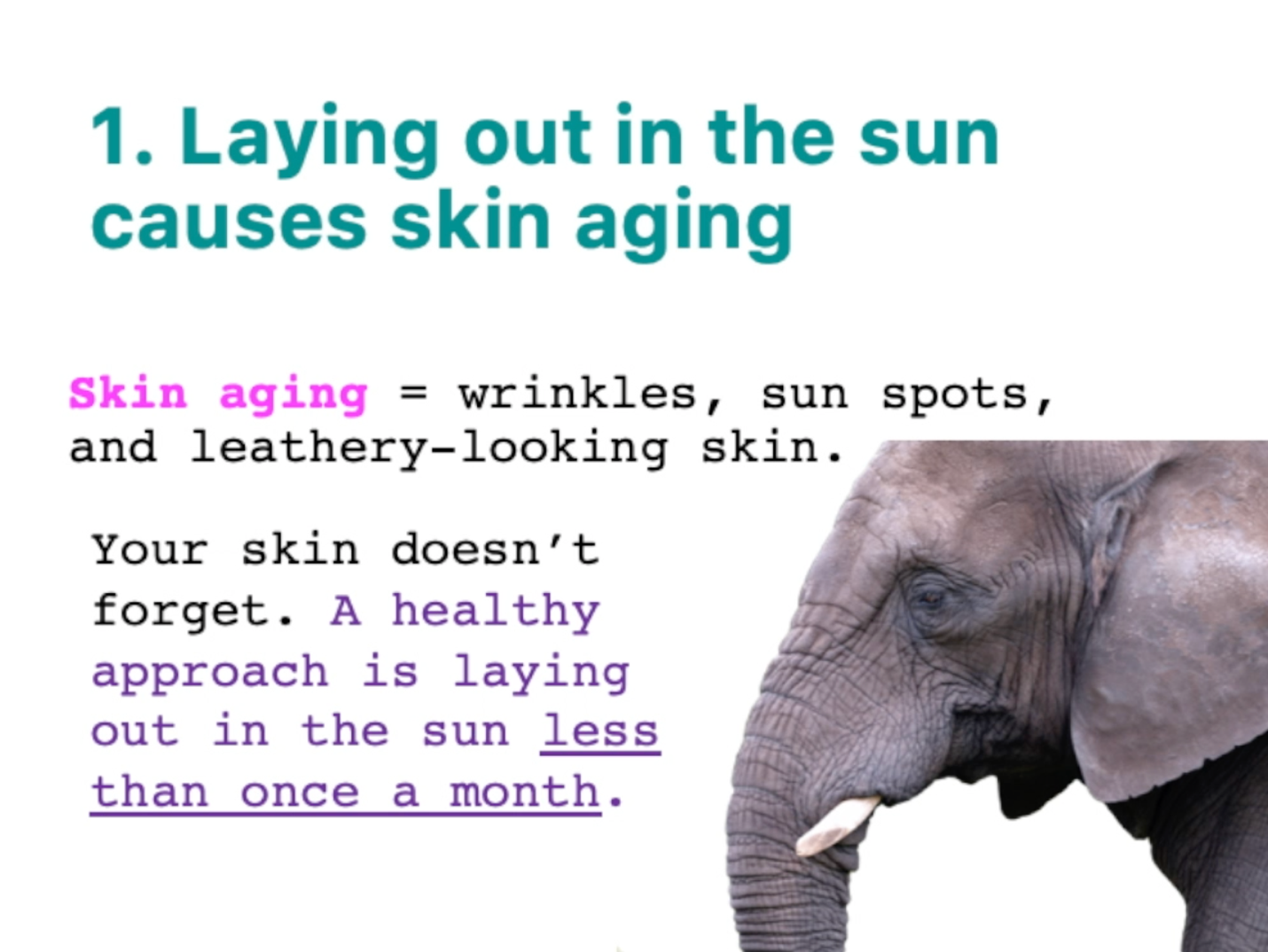

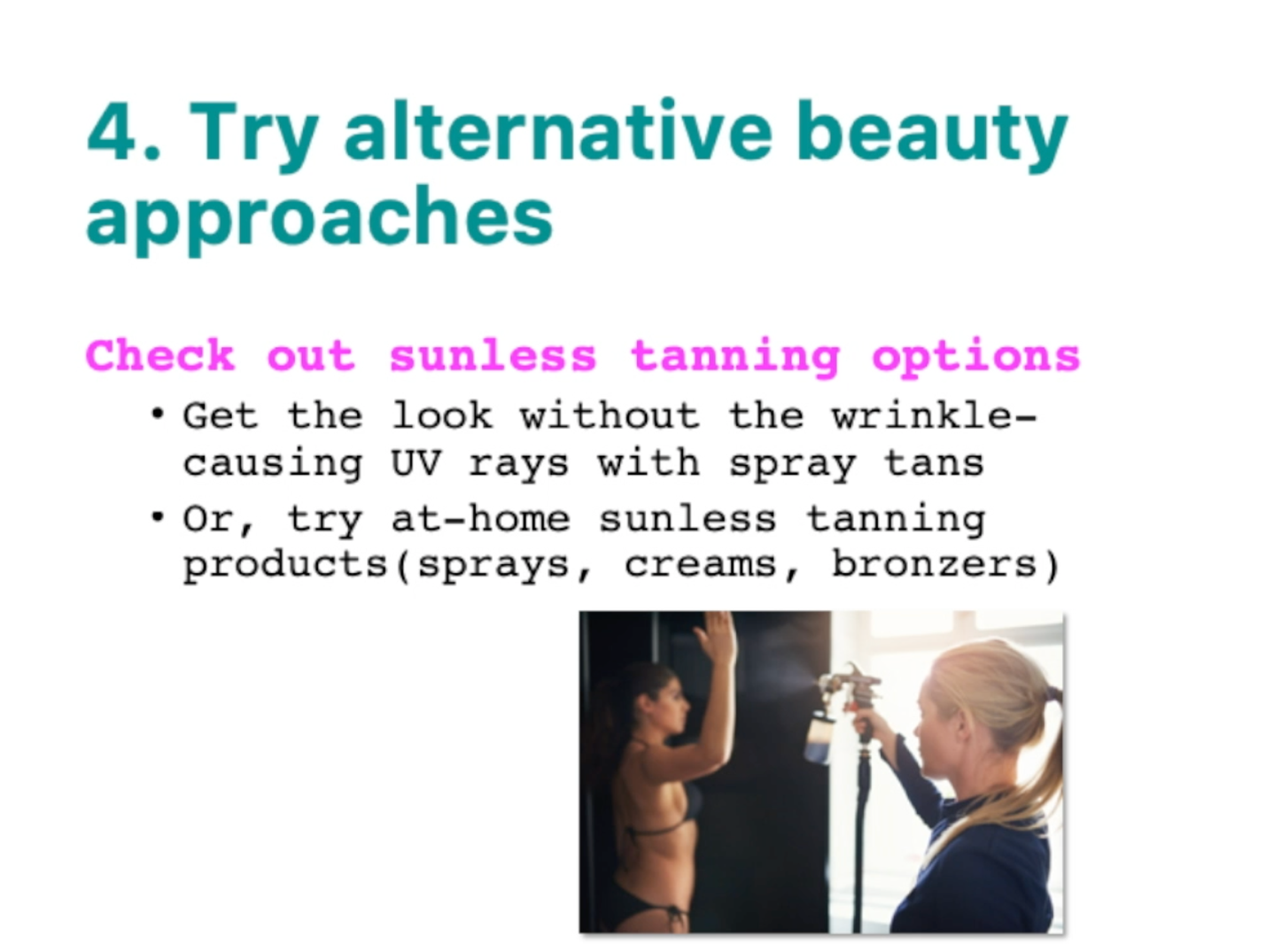

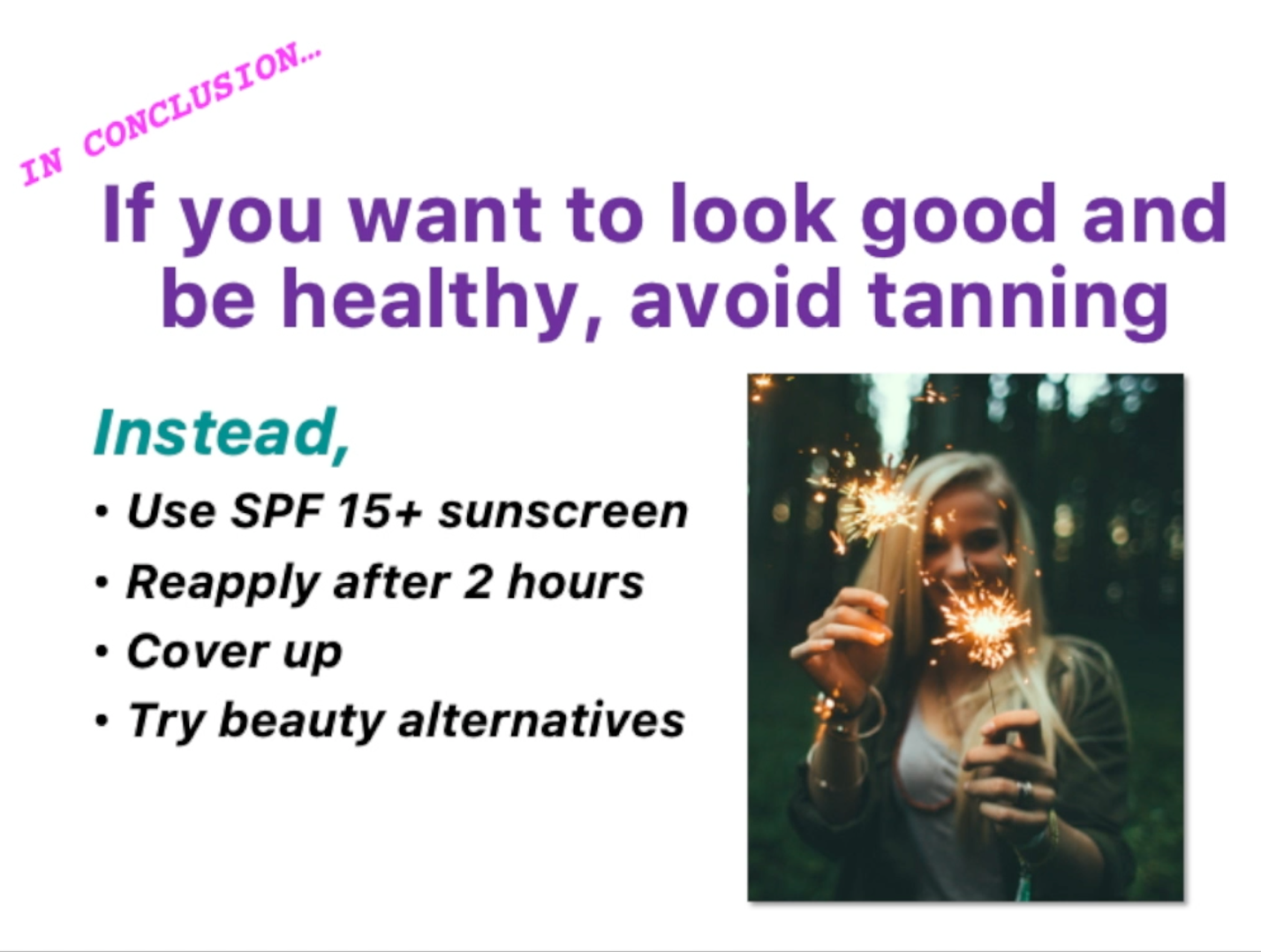


Sample text content from the Control Condition (screenshots of these locations were also included in the actual study):

---

Your Health Matters

As a Penn State student, you have a number of places on campus that can help you get healthy and stay healthy.

We’ll tell you about some of the resources you can access here on campus…”

---

University Health Services

UHS clinicians provide treatment for a wide array of health concerns that most commonly include:

- Sore throats
- Fever
- Colds
- Flu
- Asthma
- Allergies
- Routine Exams
- STIs/STDs
- And more!

---

Campus Recreation

From group exercise classes to intramural sports, Campus Recreation offers you many opportunities to be physically active. There are two buildings on campus run by Campus Recreation…

---

White Building

The White Building is located in the heart of campus, near the HUB, and offers a strength and fitness center, pool, multiple gyms, two fitness centers, a cycling studio, a fencing room, a boxing room, and a rifle/archer range.

---

IM Building

This building has a climbing suite, bouldering wall, equipment loan office where you can check out balls, rackets, and more, multiple gyms for basketball, volleyball, and badminton, and even a table tennis area.

---
