# Supplementary material for: The Effects of Embedded Skin Cancer Interventions on Sun-Safety Attitudes and Attention Paid to Tan Women on Instagram
Source: Front Psychol. 2022 Apr 8;13:838297. doi: 10.3389/fpsyg.2022.838297 (PMC9029166; doi:10.3389/fpsyg.2022.838297)
Supplement: Supplementary file 2 [file Table_2.DOCX]

**Supplementary Table**

*Bivariate correlations*

| Variable | 1. | 2. | 3. | 4. | 5. | 6. | 7. | 8. | 9. | 10. |
| --- | --- | --- | --- | --- | --- | --- | --- | --- | --- | --- |
| 1. Skin type | - |  |  |  |  |  |  |  |  |  |
| 2. App. Motivations | -.20^*^ | - |  |  |  |  |  |  |  |  |
| 3. Wave | -.17 | -.09 | - |  |  |  |  |  |  |  |
| 4. SC Condition | -.06 | -.03 | .00 | - |  |  |  |  |  |  |
| 5. AB Condition | -.07 | .05 | -.03 | -.48^***^ | - |  |  |  |  |  |
| 6. Self-compassion | .26^**^ | -.21^*^ | -.04 | -.12 | .05 | - |  |  |  |  |
| 7. Ant. Pride | .02 | -.06 | -.03 | .13 | -.02 | .44^***^ | - |  |  |  |
| 8. Norms | -.03 | .23^*^ | .02 | .11 | -.05 | .11 | -.05 | - |  |  |
| 9. Efficacy | -.07 | -.12 | .24^**^ | .11 | .08 | .17 | .39^***^ | -.18 | - |  |
| 10. Fixation Time | -.34^***^ | .04 | .18 | .12 | -.25^**^ | .02 | .00 | .10 | -.05 | - |
| 11. Sun Attitudes | -.10 | -.07 | .23^*^ | .15 | .04 | .10 | .44^***^ | -.08 | .64^***^ | -.04 |

*Note:* ^*^ *p* < .05, ^**^ *p <* .01, ^***^ *p <* .001.
